# Study on Water Suitability of Apple Plantations in the Loess Plateau under Climate Change

**DOI:** 10.3390/ijerph15112504

**Published:** 2018-11-08

**Authors:** Xuerui Gao, Ai Wang, Yong Zhao, Xining Zhao, Miao Sun, Junkai Du, Chengcheng Gang

**Affiliations:** 1 Institute of Soil and Water Conservation, Northwest A&F University, Yangling 712100, Shaanxi, China; gaoxuerui666@163.com (X.G.); gangcc@ms.iswc.ac.cn (C.G.); 2 College of Water Resources and Architectural Engineering, Northwest A&F University, Yangling 712100, Shaanxi, China; 18702929539@163.com (A.W.); sunmiao19930526@163.com (M.S.); 3 State Key Laboratory of Simulation and Regulation of Water Cycle in River Basin, China Institute of Water Resources and Hydropower Research, Beijing 100038, China; du_djk@163.com

**Keywords:** water balance, apple plantation, water suitability, climate change, the Loess Plateau

## Abstract

With the implementation of the Grain for Green Project, the apple plantation area is increasing in Loess Plateau. However, due to severe water scarcity, the sustainability of apple tree growth is threatened. In this paper, we used meteorological data (1990–2013) and forecasted climate data (2019–2050) to estimate water demand and establish a water suitability model to study the water balance between available water and water consumption of the apple trees. The results show that: (i) the order of the average water demand of apple plantation in each subarea is Shaanxi Province > Yuncheng area > Gansu Province > Sanmenxia Region, ranging from 500 to 950 mm; (ii) the temporal variability of water suitability from 1990 to 2013 is large, and the higher values are concentrated in the late growth stage of the apple trees and the lower values are concentrated in the early growth stage; (iii) the temporal and spatial distribution of water suitability is relatively stable and even in the Loess Plateau in the period of 2019–2050; (iv) the water suitability is mainly affected by effective precipitation and reference evapotranspiration and the reference evapotranspiration is mainly affected by the solar radiation (36%) and average temperature (38%). Furthermore, due to the joint influence of precipitation increases and solar radiation (average temperature) increases, the future water suitability of the apple plantation area in the Loess Plateau is showing a non-significant downward trend under RCP4.5 scenario.

## 1. Introduction

Soil erosion and water shortage are the two major bottlenecks influencing the ecological construction and sustainable economic and social development of the Loess Plateau. Since the 1990s, the Chinese government has implemented the Grain for Green Project, and the ecological construction of the Loess Plateau has resulted in significant improvements. In order to restore the ecology and increase the income of the local farmers at the same time, the Chinese government has vigorously expanded the economic forest planting areas for crops such as red date and apple in the Loess Plateau and achieved remarkable benefits. With the implementation of national policies, apple production has become an important industry for the Loess Plateau to cope with the large-scale reduction of cultivated land and to promote regional economic development effectively. It is also important to ensure the sustainable development of the Grain for Green Project. Up to 2013, under the promotion of “Northward and Westward Expansion”, the apple plantation area of Shaanxi Province in the Loess Plateau reached 950,000 hm^2^ and the output exceeded 11 million tons, accounting for one-third of the national yield [1]. The apple planting has become a leading industry solving the large-scale reduction of cultivated land effectively after the implementation of the Grain for Green Project and promotes regional economic development. Maintaining the efficient and sustainable development of the apple industry is considered a key component for the promotion of the local domestic economic income, the regional economic sustainable development, and the ecological environment. 

However, due to drought and water shortage in the Loess Plateau, the rapid development of the apple industry has changed the previous water balance. The water consumption of orchard farming under dry conditions is often greater than the water income, which causes a severe water deficit [2]. Moreover, the future climate of the area is uncertain [3]. Recent studies have shown that vegetation construction in the Loess Plateau is close to the limit of water resources [4]. In fact, the artificial plantations in this area have exceeded the deep soil water carrying capacity, resulting in deep soil desiccation [5]. In the apple tree planting areas of the Loess Plateau, the terrain is complex and the irrigation conditions are poor, therefore, the apple orchards are always under dry farming conditions. Water is an important factor for the survival of various plants; in the Loess Plateau, rainwater has become the only water resource for the development of forestry and fruit tree planting. Therefore, it is very important and necessary to carry out a macroscopic evaluation of the water suitability of apple plantations in the Loess Plateau in order to ensure the sustainable development of local apple plantations and the stable increase of farmers’ incomes.

Water suitability is the most important part of the research on the environmental suitability of plants in dry farming areas. The core of the calculation is to estimate the amount of the water demand. In recent years, some methods of measuring evapotranspiration have been proposed, but the application in large-scale areas is limited by the large uncertainties caused by the high requirement for measuring conditions [6,7]. Due to the complicated plant growth process, the estimation of water demand is difficult. Since the 1990s, many scholars have analyzed the water suitability in different growth stages of winter wheat, corn, and other economic crops based on potential evapotranspiration and precipitation [8,9,10]. The greater number of factors affecting the water demand [11] makes the estimation of evapotranspiration more difficult. Although related studies have tried to apply the water suitability estimation method of crops to fruit trees [12], research on the water suitability of apple trees in the Loess Plateau is still lacking. Especially in the context of climate change, the economic suitability of apple plantations in terms of precipitation requirements and plant water demand must be further studied in the context of future climatic conditions in the Loess Plateau in order to provide an important reference for the future economic forest planning and development in the region.

Based on this, the main purpose of this paper is to predict and estimate the water demand of apple plantations, and then to investigate the water suitability of the apple plantations in the Loess Plateau. The study first calculated the water demand of the apple orchards by estimating the potential evapotranspiration with crop coefficients (Kc). Then the observation data and the climate prediction data from coupled model inter-comparison project phase 5 (CMIP5) were used to analyze the temporal and spatial evolution of precipitation in the study area. Finally, a crop water suitability index was introduced to quantify the water suitability of apple orchards in the past and future, which could guide the planning, development, and management of apple plantation from a scientific basis. Contents of this research are as follows: (i) the Penman-Monteith formula with Kc was applied to calculate the temporal and spatial distribution of the water demand during the apple growth periods in the study area; (ii) the historical and future precipitation data in each region were used to estimate the effective precipitation of apple orchards, and then the apple water suitability estimation model was established; (iii) according to the evaluated water suitability of apple plantations in the study area, sustainable development strategies were proposed focusing on both the ecological restoration of the area and the improvement of regional farmers’ incomes. 

## 2. Materials and Methods 

### 2.1. Study Area

**Figure 1:**
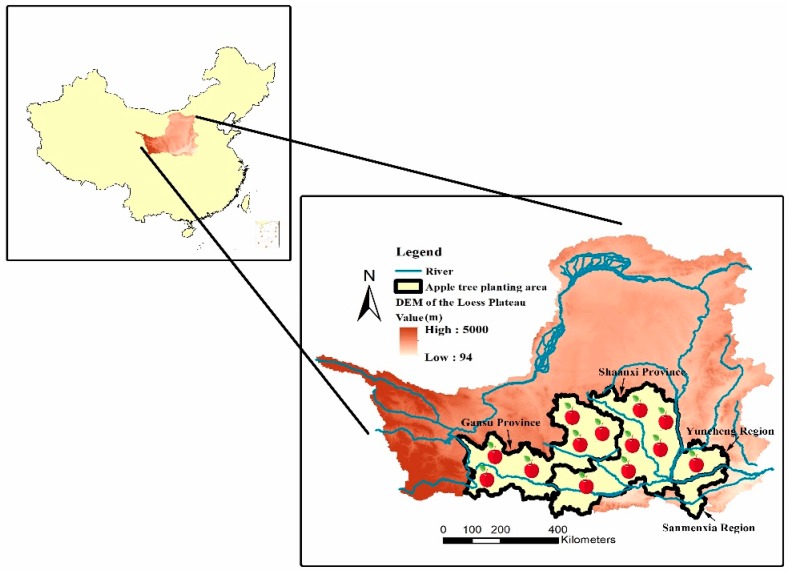
Location of suitable planting areas for apple trees in the Loess Plateau.

China is an ideal place for apple production. The four major apple producing areas include the Bohai Bay area, the Loess Plateau area, the Yellow River old channel area, and the southwest highland area. Among the four major apple producing areas, the northwest Loess Plateau area is very large and contains good quality land. As shown in Figure 1, the study area of this paper is the Loess Plateau area, including Weinan, Yan’an, Tongchuan, Xianyang, and Baoji of Shaanxi Province, Yuncheng of Shanxi Province, Sanmenxia of Henan Province, and Pingliang, Tianshui, Dingxi, and Qingyang of Gansu Province. In 2000, the total size of the apple plantations in the Loess Plateau was 847,600 hm^2^, accounting for 34.16% of China’s apple plantations. The total production output was 7.73 million tons, accounting for 38.19% of China’s apple production [13]. Under the promotion of the “Northward and Westward Expansion” layout, the apple planting area in the Loess Plateau is expected to increase continuously, accounting for one-third of the national yield, and one-eighth of the global yield. The annual average temperature of the study area is 3.6–14.3 °C, and the annual precipitation is 150–750 mm, with precipitation mainly concentrated in July to September. The annual potential evaporation is 1400–2000 mm and the annual sunshine duration is 2000 to 3100 h. Abundant sunshine radiation and long sunshine hours provide sufficient energy for photosynthesis of green plants. The long sunshine duration and high temperature difference between day and night makes the middle and southern parts of the Loess Plateau ideal locations for apple tree cultivation [14].

### 2.2. Technical Framework

**Figure 2:**
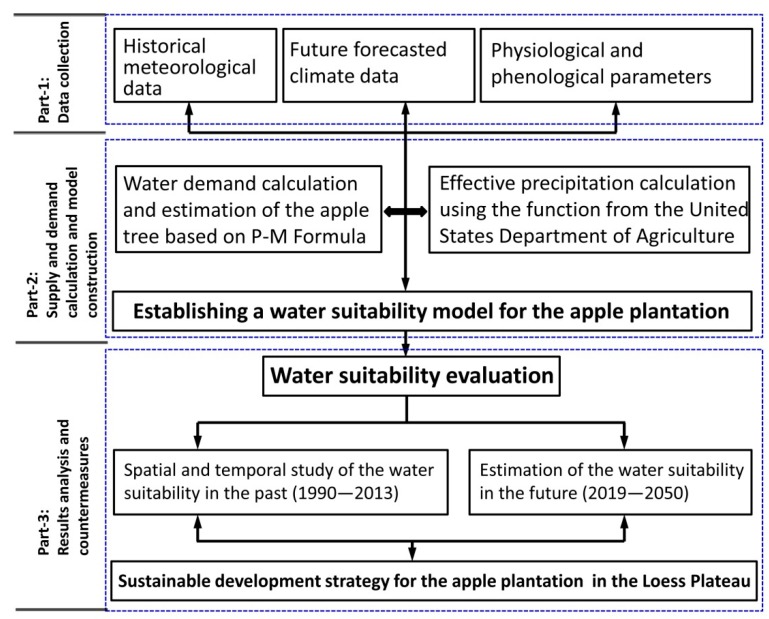
Technical roadmap of this study.

In this paper, we firstly analyzed and calculated the water demand in the apple tree growth period, and then established a water suitability model according to the effective precipitation in each sub-region. The calculated results of the model were able to quantify the water suitability of apple plantations in each sub-region. Based on the water suitability evaluation results, this paper proposed sustainable development strategies for apple plantations in the Loess Plateau in order to promote regional ecological restoration and the improvement of regional farmers’ incomes. The research framework of this paper is as shown in Figure 2.

### 2.3. Calculation of the Water Demand of Apple Trees

The water demand of apple trees in different growth stages can be calculated by following formula:

(1) $$W_{j}=\overset{n}{\underset{i=1}{\sum }}{\mathrm{Kc}}_{j}\;\times {\;\mathrm{ET}}_{0i}$$

where $W_{j}$ is the volume of water demand in the j growth stage of apple trees; ${\mathrm{Kc}}_{j}$ is the crop coefficient in the j growth stage; n is the number of ten-day periods within the whole growth period (e.g., the initial growth stage of apple trees in Gansu province is from the first ten-day period of April to the last ten-day period of May, n = 6); and ${\mathrm{ET}}_{0i}$ is the potential evapotranspiration in the i^th^ ten-day period, which can be calculated by the Penman-Monteith formula:

(2) $${\mathrm{ET}}_{0}=\frac{0.408\Delta \left(R_{n}-G\right)+\gamma \frac{900}{T+273}u2\left(e_{s}-e_{a}\right)}{\Delta +\gamma \left(1+0.34u_{2}\right)}$$

where ET_0_ is the potential evapotranspiration (mm/d); Δ is the slope of saturated water vapor pressure-temperature curve (KPa/°C); R_n_ is the net solar radiation (MJ/m^2^); G is the soil heat flux (MJ/m^2^); γ is the constant of hygrometer (KPa/°C); T is the daily average air temperature (°C); u_2_ is the wind speed at 2 m height (m/s); e_s_ is the saturated water vapor pressure (kpa); and e_a_ is the actual water vapor pressure (kpa). Due to the lack of wind speed at 2 m for most meteorological stations, the wind speed is always calculated by the formula: u_2_ = 4.78 × u_k_/ln (67.8 × h − 5.42), in which u_k_ is the wind speed at h height (m/s) and h is the observed height of the meteorological station (cm). The pressure, air temperature, water vapor pressure, relative humidity, wind speed, average value of sunshine radiation, and precipitation in a ten-day period (which is required for potential evapotranspiration calculations) were collected from the meteorological stations in study area from 1990 to 2013.

From sprout to defoliation, the growth stage of apple trees can be divided into 11 growth periods. Based on recent years of observation and experiments in meteorological stations in Henan, Shaanxi, Shanxi, and Gansu Province, the average growth periods of apple trees in sub-regions were obtained, as shown in Table 1.

Water demands of apple trees are different in different growth stages. According to the growth characteristics of apple trees in the northern Shaanxi section of the Loess Plateau, the growth period is divided into the first growth period (germination to the post-bloom), the vigorous growth period (post-bloom to 10 days after maturity), and the post-growth stage after fruit harvest (ten days after maturity to the end of the defoliation) (shown in Table 2). When calculating water suitability, it is important to select an appropriate time interval in the growth period. Many scholars prefer to choose 10 days as the time interval [12]. In the 1990s, the Food and Agriculture Organization (FAO) organized many famous scholars to conduct water demand tests on various plants (including economic crops, and fruit trees) around the world, and obtained the relationship between crop water demand and potential evapotranspiration in order to determine the Kc of various crops at different growth stages. In this study, we determined the parameters of Kc in the study areas according to research results of the FAO, which are shown in Table 2.

### 2.4. Calculation of the Water Suitability of Apple Trees

According to the ten-day water demand during the growth period of apple trees (Wi, mm) and the regional effective precipitation ($P_{e\left(dec\right)}$, mm), the ten-day water suitability of apple trees ($U_{i}$) is proposed based on the methods illustrated in the previous studies [15,16,17,18]:

(3) $$U_{i}=\{\begin{array}{cc} 1, & P_{e\left(dec\right)}\geq Wi\\ P_{e\left(dec\right)}/Wi\;, & P_{e\left(dec\right)}<Wi \end{array}$$

when the ten-day effective precipitation $P_{e\left(dec\right)}$ is larger than the water demand Wi, it means that the precipitation can satisfy the water demand, $U_{i}=1$; and when the ten-day effective precipitation $P_{e\left(dec\right)}$ is less than the water demand Wi, the ratio between them is used to represent the water suitability. Based on the above, the average suitability (U) of apple trees in the whole growth stage was estimated by the following formula:

(4) $$U=\frac{1}{n}\sum_{i=1}^{n}U_{i}$$

where *n* is the number of ten-day periods in the growth stage.

The effective precipitation is calculated by the method proposed by United States Department of Agriculture (USDA) [19]:

(5) $$P_{e\left(dec\right)}=\{\begin{array}{ll} P_{dec}\times (125-0.6\;\times \;P_{dec})/125 & ,\;P_{dec}\leq \left(\frac{250}{3}\right)\mathrm{mm};\\ 125/3+0.1\;\times \;P_{dec} & ,\;P_{dec}>\left(\frac{250}{3}\right)\mathrm{mm} \end{array}$$

where $P_{e\left(dec\right)}$ is the effective precipitation in the ten-day period; $P_{dec}$ is the total precipitation observed in the ten-day period, which can be collected from the meteorological station, mm; the effective precipitation of the whole growth period is the sum of effective precipitation within each ten-day period.

### 2.5. Predication Method of Future Climatic Factor

This paper used the global climate data in the fifth report published by the Intergovernmental Panel on Climate Change (IPCC) and the corresponding regional downscaling method to analyze the climate elements in the future (2019–2050). The projected climate change data for the study area in the Loess Plateau from 2019 to 2050 are from the World Climate Research Programmer’s (WCRP) coupled model inter-comparison project phase 5 (CMIP5) multi-model dataset. The multi-model dataset is produced by a simple averaging method based on 21 global climate system models. Table 3 lists the basic information of the 21 Global Climate Models (GCM) and the associated institutions. Based on the GCM data, the National Climate Center of China used RegCM4.0 for downscaling and obtained the projected climate data of different scenarios from 2019 to 2050 in the Loess Plateau with a spatial resolution of 0.5°. The projected climate data mainly include daily precipitation, daily mean, maximum and minimum temperature, daily mean relative humidity, daily mean atmospheric pressure, daily mean radiation intensity, and the daily mean wind speed. It must to be noted that CMIP5 divides the future greenhouse gas emission scenarios into four types (RCP2.6, RCP4.5, RCP6.0, and RCP8.5), and they are called the Representative Concentration Pathway [20]. In the four RCP scenarios, RCP4.5 radiative forcing is stable at 4.5 W/m^2^. After 2100, the CO_2_ equivalent concentration is stable at about 650 × 10^−6^ [21]. In order to limit greenhouse gas emissions, it is necessary for countries worldwide to change their current energy systems and use more electric energy and low-emission energy technologies. Comparatively, the RCP4.5 scenario is a moderate scenario that is always used for future climate change prediction. In this paper, we therefore analyzed the precipitation, potential evapotranspiration, and water suitability on the Loess Plateau based on the RCP4.5 scenario.

### 2.6. Contribution Rate and Sensitivity Analysis Method

In addition to precipitation, the factors influencing the reference evapotranspiration (ET_0_) are imposing substantial impacts on the water suitability of apple trees. Based on the Penman-Monteith formula, the change of ET_0_ can be attributed to the change of radiation and the elements of aerodynamics, which are the results of the changes in wind speed (U_2_), solar radiation (R_n_), average air temperature (T_mean_), and actual water vapor pressure (e_a_). Therefore, the partial derivatives of ET_0_ to wind speed (U_2_), solar radiation (R_n_), average air temperature (T_mean_), and actual water vapor pressure (e_d_) are calculated as follows [42]:

(6) $$\frac{\partial ET_{0}}{\partial U_{2}}=\frac{\gamma \frac{900}{T_{mean}+273}U_{2}\left(e_{s}-e_{a}\right)\Delta +\gamma \left(1+0.34U_{2}\right)-0.34\gamma \;\times \;(0.408\Delta \left(R_{n}-G\right)+\gamma \frac{900}{T_{mean}+273}U_{2}\left(e_{s}-e_{a}\right)}{{\left(\Delta +\gamma \left(1+0.34U_{2}\right)\right)}^{2}}$$

(7) $$\frac{\partial ET_{0}}{\partial R_{n}}=\frac{0.408\cdot \Delta }{\Delta +\gamma \left(1+0.34U_{2}\right)}$$

(8) $$\begin{array}{ll} \frac{\partial ET_{0}}{\partial T_{mean}}= & \frac{\left(\frac{\partial \left(0.408\Delta \left(R_{n}-G\right)\right)}{\partial T_{mean}}+\frac{\partial \left(\gamma \frac{900}{T_{mean}+273}U_{2}\left(e_{s}-e_{a}\right)\right)}{\partial T_{mean}}\right)\left(\Delta +\gamma \left(1+0.34U_{2}\right)\right)}{\Delta +\gamma \left(1+0.34U_{2}\right){)}^{2}}-\\ & \frac{\frac{\partial \Delta }{\partial T_{mean}}\times (0.408\Delta \left(R_{n}-G\right)+\gamma \frac{900}{T_{mean}+273}U_{2}\left(e_{s}-e_{a}\right)}{{\left(\Delta +\gamma \left(1+0.34U_{2}\right)\right)}^{2}} \end{array}$$

(9) $$\frac{\partial ET_{0}}{\partial \left(e_{s}-e_{a}\right)}=\frac{0.408\Delta \;\times \;\frac{\partial R_{n}}{\partial \left(e_{s}-e_{a}\right)}-\gamma \frac{900}{T_{mean}+273}U_{2}}{\Delta +\gamma \left(1+0.34U_{2}\right)}$$

The meanings of the above symbols can be found in the previous equations. Based on the above equations, the contribution of meteorological factors to the change of *ET*_0_ can be calculated as the following total derivative equation [43]:

(10) $$d\mathrm{ET}0_{\;}=\;\frac{\partial {\mathrm{ET}}_{0}}{\partial U_{2}}\cdot dU2\;+\;\frac{\partial {\mathrm{ET}}_{0}}{\partial R_{n}}\cdot dR_{n}+\frac{\partial {\mathrm{ET}}_{0}}{\partial T_{\mathrm{mean}}}\cdot dT_{\mathrm{mean}}+\frac{\partial {\mathrm{ET}}_{0}}{\partial e_{d}}\cdot d\left(e_{s}-e_{a}\right)$$

Therefore, the temporal changing rate of ET_0_ is shown as:

(11) $$\frac{dET_{0}}{dt}=\frac{\partial ET_{0}}{\partial U_{2}}\frac{dU_{2}}{dt}+\frac{\partial ET_{0}}{\partial R_{n}}\frac{dR_{n}}{dt}+\frac{\partial ET_{0}}{\partial T_{mean}}\frac{dT_{mean}}{dt}+\frac{\partial ET_{0}}{\partial (e_{s}-e_{a)}}\frac{d(e_{s}-e_{a)}}{dt}$$

where the left side is the change of ET_0_ over the study period and the right side is the actual contribution rate of wind speed (U_2_), solar radiation (R_n_), average air temperature (T_mean_), and actual water vapor pressure (e_a_).

The calculation of the sensitivity coefficient of ET_0_ to a certain meteorological factor x is as follows [44]:

(12) $$S\left(x\right)=\frac{\partial ET_{0}}{\partial x}\;\times \;\frac{x}{ET_{0}}$$

where S(x) represents the sensitivity coefficient and x is one of the meteorological factors. A positive value of the sensitivity coefficient indicates that ET_0_ will increase as the unit of the meteorological factor increases.

## 3. Results

### 3.1. Spatial-Temporal Evolution Characteristics of Water Demand of Apple Trees in the Past Period

**Figure 3:**
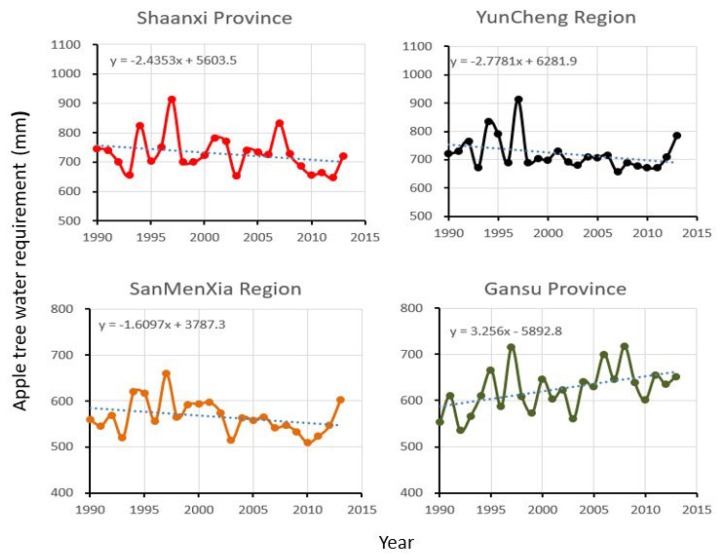
Temporal evolution of water demand of apple trees in each province from 1990 to 2013.

Water is often the most important factor for plant growth in arid and semi-arid regions. Usually, the potential evapotranspiration of plants is used to represent the water demand of the plant or crop. The Kc is used to estimate the water demand of the apple trees during the whole growth period in this study. Figure 3 shows the calculated water demands of apple trees in the four sub-areas in the past period (1990–2013).

The order of average annual water demand of apple trees in the four sub-areas is Shaanxi Province > Yuncheng District > Gansu Province > Sanmenxia Region, which are respectively 729 mm, 722 mm, 624 mm, and 566 mm. Upon viewing the graphs of the temporal changes in Figure 3, it can be observed that the changing trends of the annual water demand of the apple trees in the four regions from 1990 to 2013 are different. The water demands of apple trees in Shaanxi Province, Yuncheng Region, and Sanmenxia Region all show a decreasing trend in the past period. However, the water demand of apple trees in Gansu Province shows an obvious increasing trend. This is mainly related to the variability of the geographical and climatic factors.

**Figure 4:**
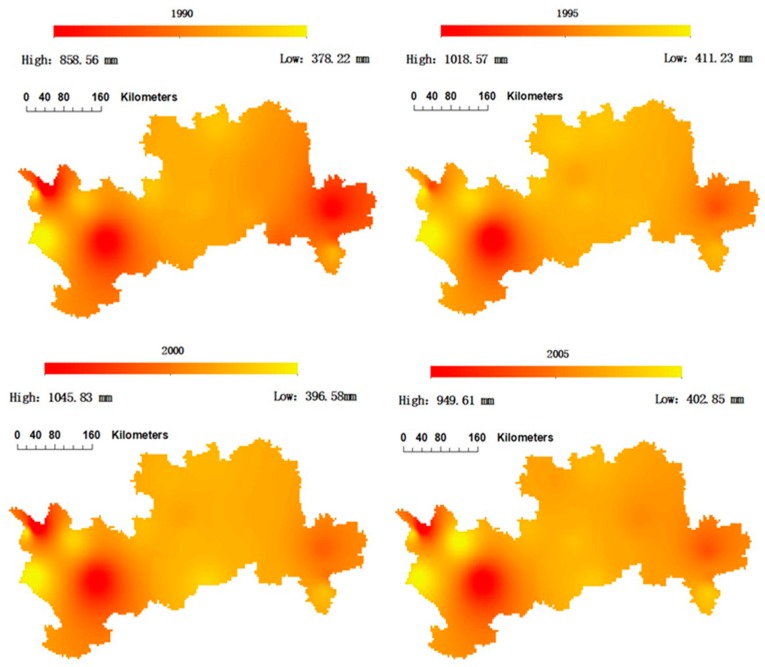

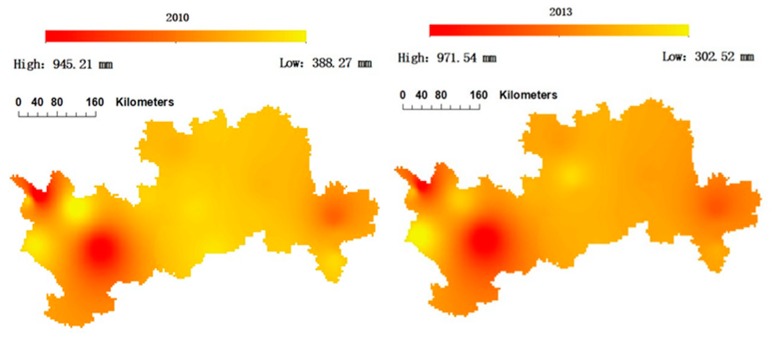
Spatial evolution of the water demand of apple trees in each province from 1990 to 2013.

Figure 4 shows the spatial distribution of the average water demand of apple trees in the four sub-areas from 1990 to 2013. It was found that the water demand of the apple trees was relatively evenly distributed in the north-south direction, and the values from the east and the west were relatively larger than the middle. The high value areas of apple tree water demand were concentrated in Yuncheng Region and Tianshui and Dingxi Regions of Gansu Province. The average annual water demand was higher than 500 mm and the highest was around 1000 mm. A slight decreasing trend in the water demand of apple trees can be observed, especially for the middle and east areas, which may have resulted from the climate changes in recent years leading to a significant decline in the potential evapotranspiration of apple trees. Therefore, it is important to study the potential evapotranspiration of apple trees under future climate change conditions and then forecast the gap between water supply and demand of apple plantations.

### 3.2. The Spatial-Temporal Evolution of Water Suitability in the Past Period

The Loess Plateau is located in a semi-arid region with insufficient surface water resources. Many fruit trees are planted in mountainous areas without irrigation conditions, and the water resources are quite limited. Therefore, the water required for plant growth during the whole growth period depends on atmospheric precipitation. To simplify the description of the temporal evolution of apple tree water suitability, the study area was divided into the eastern part of the study area (Yuncheng and Sanmenxia Regions), the western part of the study area (Dingxi, Pingliang, and Tianshui Regions), the southern part of the study area (Baoji, Xianyang, Tongchuan, and Weinan Regions), and the northern part of the study area (Qingyang and Yan’an Regions). Based on the results, it was found that the water suitability of apple trees in each growth period is not exactly the same as the whole growth period. Generally, the water suitability in the initial growth period is the lowest, and the highest values are concentrated in the later stage of growth, which is mainly because the precipitation in the Loess Plateau is mainly concentrated in the summer and autumn months. We also found that the main contradiction between water supply and water demand for apple plantations in the Loess Plateau is the spring drought, which represents a potential threat for vegetation restoration. Moreover, we further analyzed the temporal change trend of water suitability of each study area in the past period (1990–2013).

**Figure 5:**
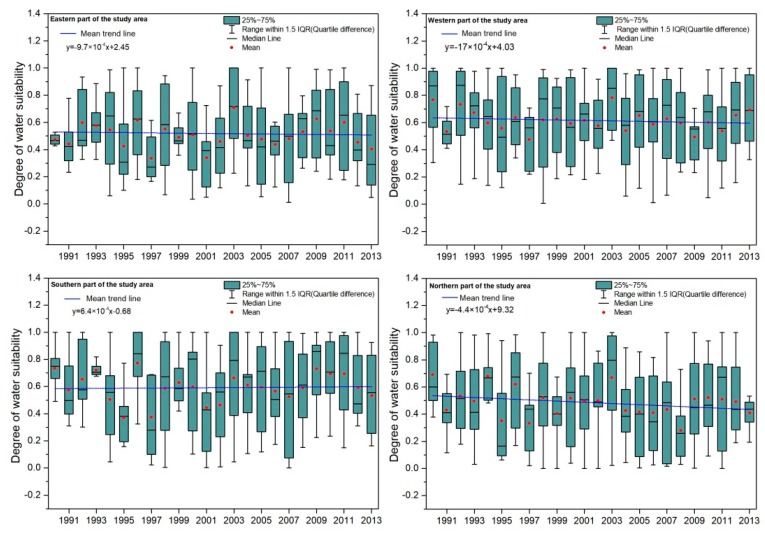
Temporal evolution of water suitability of apple trees from 1990 to 2013.

In Figure 5, the variation is large according to the mean value of the water suitability. The highest water suitability in the eastern part of the study area was in 2003, with about 50% of the values above 0.5, and the lowest value appeared in 1997, with 50% of the values below 0.5. The highest water suitability in the western part of the study area appeared in 2003, with the mean value around 0.8, and the minimum appeared in 1997. The maximum value of water suitability in the southern part of the study area appeared in 1996, and 50% of the values concentrated at 0.65 or larger. The minimum value appeared in 1997, with the mean value of suitability less than 0.4. The mean values of water suitability in northern part of the study area were the lowest of all the study areas. Overall, the slopes of the changing water suitability trends are all negative except for the southern part of the Loess Plateau, which indicates that the water suitability was showing an obvious downward trend in the past period of 1990–2013. The maximum water suitability was always concentrated in September and October, which represent the later growth stages of the apple trees. The minimum water suitability was usually concentrated in the early growth stages for apple trees in March and April. Our results are consistent with the previous research of Yang Xiaoli [12].

**Figure 6:**
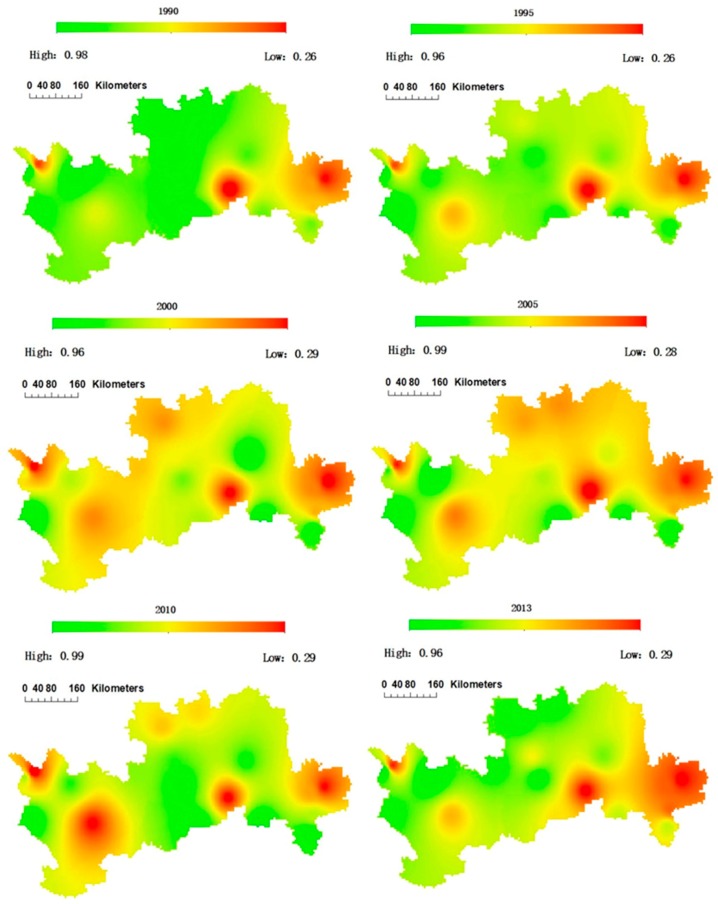
Spatial evolution of the water suitability of apple trees in the study area from 1990 to 2013.

Table 4 shows the multi-year average and root mean square error of water demand, effective precipitation, and water suitability for each study area. From the perspective of the whole growth period, the effective precipitation is much smaller than the water demand, and the annual average of effective precipitation and water demand varies greatly. It can be seen from the table that the water suitability in the western part of the study area is the best, and the water suitability in the eastern part is the worst, indicating that the water scarcity in the eastern part of the study area is much more severe. The spatial evolution of apple tree water suitability is further analyzed as shown in Figure 6.

Figure 6 shows the spatial distribution of water suitability of apple trees from 1990 to 2013. The results show that the order of the water suitability is Sanmenxia Region (southern region) > Shaanxi Province (northern part) > Gansu Province (western part) > Yuncheng (eastern part). The low-value areas are located in Yuncheng, Tongchuan of Shaanxi Province, and Tianshui of Gansu Province. This may be due to the larger water demand of apple trees and the relatively lower rainfall in these areas. In general, Shaanxi Province is the main apple production area in China, whose apple production accounts for more than 30% of the national total. In this study, we found that the water suitability of apple trees in Shaanxi Province (north and middle area of the Loess Plateau) is relatively higher compared with other areas. 

### 3.3. The Water Suitability under Future Climate Change

The IPCC 5th Assessment Report points out that average global temperatures haves been increasing since the second half of the 19th century, and the average temperatures of the past three decades was higher than before. The report also pointed out that temperatures at 0–700 m above sea surface showed a positive trend in the period 1971–2000, and the ocean heat content also increased [45]. The report further confirmed the fact of climate warming in recent years through new scientific observations, more sophisticated attribution analysis, and climate system models. Based on this trend, this paper analyzed the changes of water suitability of apple trees in the study area from 2019 to 2050 under the RCP4.5 scenario. 

**Figure 7:**
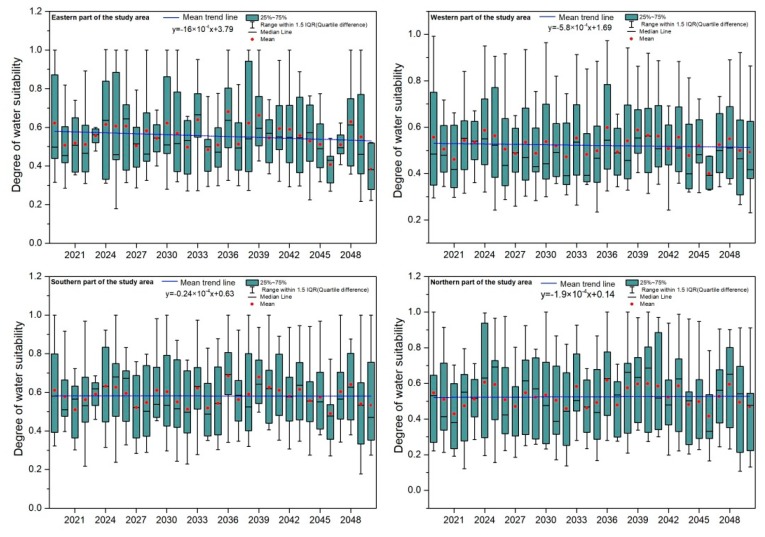
Temporal evolution of water suitability of apple trees in each province from 1990 to 2013.

As can be seen in Figure 7, the water suitability of apple plantations in the four study areas is projected to be relatively stable in the period of 2019 to 2050, and the mean values are all projected to be concentrated in the range of 0.4 to 0.7. It should be noted that the maximum values of water suitability in the four sub-regions all appear in 2036, which indicates that the spatial distribution of water suitability in the future is even in the Loess Plateau. We also found that although the projected changing trend of water suitability is generally showing a decreasing trend under the climate change scenarios, the slope of the changing trend is reduced compared with the past period (1990–2013). Therefore, we speculate that the water available for apple tree growth will be more sufficient in the future. Because climate change imposes great impacts on precipitation and evapotranspiration and thus influences the water demand and the water suitability of apple trees, it is necessary to pay attention to the impact of climate change on water suitability of apple plantations in the future.

Table 5 shows the future multi-year average and root mean square error of water demand, effective precipitation, and water suitability in each sub-region. Compared with the past period (1990–2013), the water suitability in the future (2019–2050) is projected to be generally higher (except the western part), indicating that with the climate change the contradiction between water supply and demand will be alleviated.

**Figure 8:**
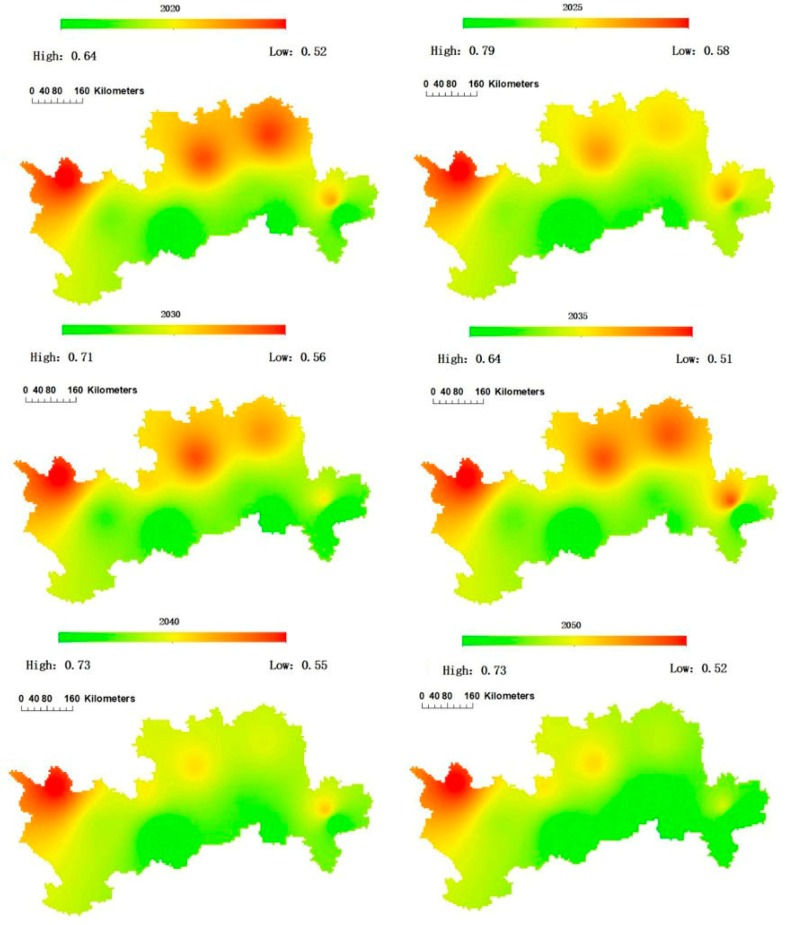
Spatial evolution of the water suitability of apple trees in each province from 2019 to 2050.

Figure 8 shows the projected spatial evolution of water suitability of apple trees in each study area from 2019 to 2050. The projected water suitability of apple trees in the four sub-areas increases as the longitude increases, but it decreases as the latitude increases. Generally, the northern and western parts are projected to be poor for water suitability compared with the eastern and southern parts. In 2020, the average value of water suitability in the study area is projected to be only 0.56, but in 2040 and 2050, it is estimated to be 0.63 and 0.66. Consequently, the sustainability of apple plantations is estimated to be better in the future under climate change compared with the past period due to precipitation increases and potential evapotranspiration decreases.

## 4. Discussion

### 4.1. The Comparison between Our Findings and Those of the Previous Studies

In order to evaluate the reliability of the results of this study, we compared the results of this study with previous studies. By analyzing the water suitability of apple orchards in the past, we found that the water suitability of apple trees at different growth stages is not completely consistent within the whole growth period. A previous related research study arrived at similar conclusions to our own. Yang et al. found that apple trees had poor water suitability in the initial growth period, that the water requirement after apple ripening was lower than the initial growth period, and that the water suitability reached the highest value in the apple ripening stage [12]. Our study also calculated the future water suitability of apple trees through analyzing the water balance based on the estimation of future climate elements, in which the global climate data released by the IPCC Fifth Research Report and the corresponding regional downscaling methods were used. Previous related research studies had a similar approach to our own. Liu et al. used 19 GCMs under RCP4.5 scenario derived from CMIP5 and it was also found that the results were reliable [46]. Zhu et al. evaluated the reliability and accuracy of precipitation data generated by GCMs [47]. Gao et al. analyzed the future climate data released by CMIP5 based on regional downscaling model (RegCM4.0) and found that the precipitation is expected to increase significantly (5% confidence level) in the Loess Plateau under both the RCP2.6 and RCP8.5 scenarios [48]. In the above studies, the methods used are close to those used in our study and the findings are also consistent with our results.

### 4.2. The Changing Characteristics of the Contributing Factors for Water Suitability

**Figure 9:**
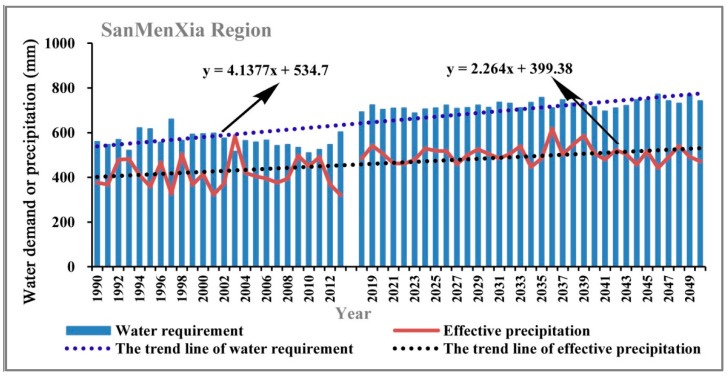
The changing trend of the water demand of apple trees and the effective precipitation in Sanmenxia Region in the periods of 1990–2013 and 2019–2050.

**Figure 10:**
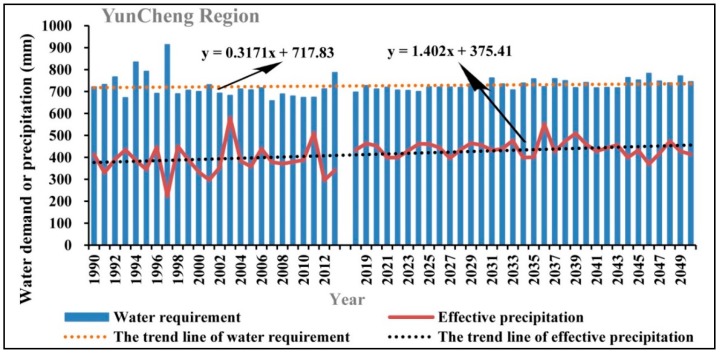
The changing trend of the water demand of apple trees and the effective precipitation in Yuncheng Region in the periods of 1990–2013 and 2019–2050.

**Figure 11:**
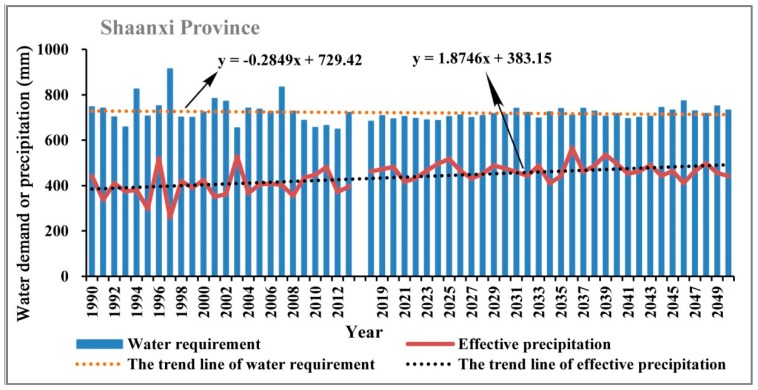
The changing trend of the water demand of apple trees and the effective precipitation in Shaanxi Province in the periods of 1990–2013 and 2019–2050.

**Figure 12:**
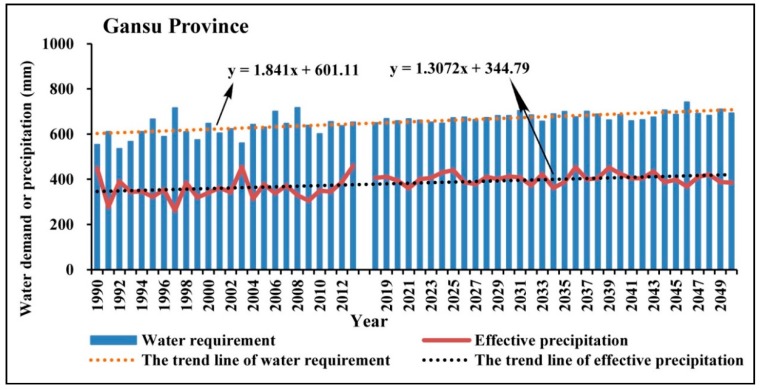
The changing trend of the water demand of apple trees and the effective precipitation in Gansu Province in the periods of 1990–2013 and 2019–2050.

Water suitability is proposed as the ratio of precipitation and water demand in each growth stage of apple trees through the estimation of water demand in each stage of growth using Penman-Monteith formula based on the observed meteorological data during the growth period of apple trees. Therefore, the potential influencing factors mainly include precipitation, temperature, humidity, solar radiation, wind speed, and so on. Firstly, we used the historical observed data and the climatic forecasted data to analyze the changing characteristics of water demand for apple trees in the study area, as shown in Figure 9, Figure 10, Figure 11 and Figure 12.

As can be seen in Figure 9, Figure 10, Figure 11 and Figure 12, during the period of 1990–2050, the apple tree water demand values in the four sub-areas are all greater than the effective precipitation, therefore the water requirement for apple tree growth cannot be fully satisfied in the Loess Plateau. However, notable differences can be observed among different sub-regions in the Loess Plateau in regard to the changing characteristics of water requirements and effective precipitation. In Sanmenxia Region and Gansu Province, the water requirement and effective precipitation are both showing an obvious growing trend in the period of 1990–2050, but the growth rate of the water requirement is significantly larger than the growth rate of effective precipitation. Comparatively, in Yuncheng Region and Shaanxi Province, the growth rate of the water requirement is lower than the growth rate of effective precipitation. The above findings indicate that the water balance in the core part of the suitable area for apple tree growth in the Loess Plateau is much better than other areas.

### 4.3. Analysis of the Contribution Rate and Sensitivity of Various Meteorological Factors to ET_0_

Based on the method outlined in Section 2.6, we calculated the contribution rate of four meteorological factors to $ET_{0}$ and the sensitivity coefficient of $ET_{0}$ to wind speed, average temperature, solar radiation, and actual vapor pressure. The calculation results are shown in Table 6.

The contribution rates of the four meteorological factors to $ET_{0}$ were calculated, which are 18% (U_2_), 36% (R_n_), 38% (T_mean_), and 8% (e_a_), respectively. It was found that the temperature, solar radiation, and the effective precipitation are the main factors influencing the water suitability of apple plantations in the Loess Plateau. Generally, the sensitivity coefficient of $ET_{0}$ to actual water vapor pressure (which is −0.53) is the highest among the four sensitivity coefficients, the sensitivity coefficient to solar radiation and average temperature is second and third, 0.46 and 0.34, respectively, and the sensitivity coefficient to wind speed is the smallest (only 0.27). The above results show that if the solar radiation, the average temperature, and the wind speed are reduced by 10%, the $ET_{0}$ will decrease by 4.6%, 3.4%, and 2.7%, respectively. Moreover, a 10% reduction in actual vapor pressure will result in an increase of $ET_{0}$ at 5.3%.

### 4.4 The Sustainable Development Strategy of the Apple Plantations in the Loess Plateau

#### 4.4.1. The Adoption of the “Water-Vegetation Harmony” Management Concept

The Loess Plateau is poor in water resources. The rivers and reservoirs within the Loess Plateau are always at low elevation, while apple trees are planted on hillsides, therefore, irrigation is very difficult, and precipitation is almost the only source of growth for apple plantations in the region. Due to the deep aerated zone in the Loess Plateau, the soil water resources represent a key form of support for vegetation restoration and ecological development, which is also the key linkage between surface water, groundwater, and crop water. According to a previous study, the soil water in the Loess Plateau accounts for more than 80% of the potential rainwater resources in the arid and semi-arid areas of China [49]. As a consequence, we propose the “water-vegetation harmony” management concept, which means that the planting structure of apple plantations in the Loess Plateau should be strictly determined by the soil water resources carrying capacity. 

The concept of “water-vegetation harmony” management is derived from the concept of optimum water agriculture, which means pursuing the balance between the demand and supply of agricultural water resources through various measures in order to improve the matching degree of crop water demand and local available water resources. This study provides an important method for the “water-vegetation harmony” management based on the index of water suitability, which can be widely applied in other similar research studies in the future.

#### 4.4.2. The Integration of the Rainwater Harvesting and Agronomic Measures into a New Technical Mode for Vegetation Ecological Construction in Arid and Semi-Arid Areas

Soil water and rain water are the main forms of water resources in the Loess Plateau. Therefore, to achieve sustainable development of the vegetation ecosystem and agricultural production system, it is very necessary to tap the soil water potential and increase the rainwater harvesting and utilization rate. It is also necessary to promote agronomic measures, to strengthen modern farming management on dry lands, and to develop rainwater collecting technologies, high-efficiency and precision irrigation technologies, soil moisture prediction technologies, and modern wisdom agricultural management technologies. More importantly, a new technical model should be established to promote comprehensive water-saving technologies and management strategies.

#### 4.4.3. The Establishment of a Reasonable Rewards and Penalties Systems to Ensure the Sustainable Development of Apple Plantations in the Loess Plateau

The Grain for Green Project in the Loess Plateau is not only a regional project, but also a national project in China, and it is not only a public ecological protection project, but also a huge economical investment project. Therefore, it is related to the sustainable development of all Chinese people. In recent years, the Grain for Green Project has achieved remarkable results mainly because it relies on national policy subsidies with huge economic inputs. Thus, the continuous economic inputs represent the decisive factor to the development of vegetation in the region. Based on this, in order to maintain the sustainable development of vegetation on the Loess Plateau and to improve the function of the Grain for Green Project, a national compensation system that uses government transfer payments to ask the developed area to compensate the developing areas in the Loess Plateau is urgently needed. In addition, a performance appraisal system for basic-level government should be established as soon as possible. Under this system, the regional government(s) that performs well can be rewarded and the regional government(s) that perform poorly can be warned and/or punished.

## 5. Conclusions

With the implementation of Grain for Green Project in the Loess Plateau, apple production is becoming one of the dominating industries for the local economy. However, due to severe water scarcity, the sustainability of apple tree growth is threatened. To evaluate the sustainability of apple tree development in the Loess Plateau, this paper established a water suitability model for apple plantations and analyzed the apple tree water suitability in each of the sub-regions. The main findings are as follows:

(1) In the past period (1990–2013), the order of the average water demand of apple trees in each sub-region was Shaanxi Province > Yuncheng Region > Gansu Province > Sanmenxia Region, and the water demand ranged from 500 to 950 mm. The temporal variability of water suitability from 1990 to 2013 was large, and the higher values of water suitability were concentrated in the late growth stage of the apple trees and the lower values were concentrated in the early growth stage of the apple trees.
(2) In the future (2019–2050), the temporal evolution of apple tree water suitability is projected to be relatively stable, and the spatial distribution is projected to be relatively even. Generally, the water suitability in the northern and western areas is expected to be lower than that in the southern and eastern areas.
(3) We found that the water suitability is mainly affected by effective precipitation and meteorological factors including wind speed (U_2_), solar radiation (R_n_), average temperature (T_mean_), and actual vapor pressure (e_a_). The contribution rates of the four factors to ET_0_ change are respectively 18%, 36%, 38%, and 8%.
(4) The findings in this paper indicate that the water suitability of apple plantations showed a decreasing trend in the past period (1990–2013) but that the slope of downward trend of water suitability is expected to be greatly reduced in the future period. Therefore, we speculate that the water available for apple tree growth will be more sufficient under future climate change than that observed in the past period. However, with that said, it is important not to be over-optimistic. With this in mind, we also proposed several key measures to improve the sustainability of apple plantations in the Loess Plateau in the future.

## Figures and Tables

**Table 1:** The average growth period of apple tree in different subareas (month-day).

Growth Period	Henan	Shaanxi	Shanxi	Gansu
Germination	03-08	03-11	03-18	04-07
Green Tip	03-12	03-15	03-22	04-11
Half-Inch Green	03-20	03-24	03-30	04-16
Pink	04-01	04-06	04-14	04-24
Full Bloom	04-13	04-17	04-19	04-27
Post-Bloom	04-22	04-28	04-27	05-04
Fruit Present	10-02	10-08	10-09	09-17
Beginning of leaf	10-27	10-31	10-30	10-02
End of leaf	11-04	11-10	11-09	10-09
First Defoliation	11-10	11-15	11-10	10-12
Last Defoliation	11-25	11-28	11-25	11-17

**Table 2:** Growth stages and crop coefficients of apple trees.

Province	First Growth Period	Vigorous Growth Period	Post Growth Period
Henan	First ten-day period of Mar.–Last ten-day period of Apr.	First ten days of May–Last ten days of Oct.	Second ten-day period of Oct–Last ten-day period of Nov.
Shanxi	Second ten-day of-Mar–Last ten-day of Apr	First ten -days of May–Last ten days of Oct.	Second ten-day period of Oct–Last ten-day period of Nov.
Shaanxi	Last ten-day period of Mar.–Last ten-day period of Apr.	First ten days of May–Last ten days of Oct.	Second ten-day period of Oct.–Last ten-day period of Nov.
Gansu	First ten-day period of Mar.–First ten-day period of Apr.	Second ten-day period of May–Second ten-day period of Oct.	Last ten-day period of Oct.–Second ten-day period of Nov.
Kc	0.55	0.90	0.65

**Table 3:** Summary of 21 general circulation models from coupled model inter-comparison project phase 5 (CMIP5).

Model	Institution	Resolution	References
BCC-CSM1.1	Beijing Climate Center, Meteorological Administration	128 × 64	Xin et al. (2013) [22]
BNU-ESM	Beijing Normal University, China	128 × 64	Ji et al. (2014) [23]
CanESM2	Canadian Centre for Climate Modelling and Analysis, Canada	128 × 64	Chylek et al. (2011) [24]
CCSM4	National Center for Atmospheric Research, USA	288 × 192	Subramanian et al. (2012) [25]
CNRM-CM5	Centre National de Recherches Meteorologiques, Météo-France, France	256 × 128	Voldoire et al. (2013) [26]
CSIRO-MK-3.6.0	Australian Commonwealth Scientific and Industrial Research Organization, Australia	192 × 96	Rotstayn et al. (2013) [27]
FGOALS-g2	Institute of Atmospheric Physics, Chinese Academy of Sciences, China	128 × 60	Zhou et al. (2013) [28]
FIO-ESM	The First Institution of Oceanography, SOA, China	128 × 64	Qiao et al. (2013) [29]
GFDL-CM3	NOAA Geophysical Fluid Dynamics Laboratory, USA	144 × 90	Donner et al. (2011) [30]
GFDL-ESM2G	NOAA Geophysical Fluid Dynamics Laboratory, USA	144 × 90	Dunne et al. (2012) [31]
GFDL-ESM2M	NOAA Geophysical Fluid Dynamics Laboratory, USA	144 × 90	Dunne et al. (2012) [31]
GISS-E2-H	NASA Goddard Institute for Space Studies, USA	144 × 90	Shindell et al. (2013) [32]
GISS-E2-R	NASA Goddard Institute for Space Studies, USA	144 × 90	Schmidt et al. (2010) [33]
HadGEM2-AO	National Institute of Meteorological Research, Korea Meteorological Administration, Seoul, South Korea	192 × 145	Baek et al. (2013) [34]
IPSL-CM5A-LR	Institut Pierre-Simon Laplace, France	96 × 96	Dufresne et al. (2013) [35]
MIROC5	Tokyo, and National Institute for Environmental Studies (Japan)	256 × 128	Watanabe et al. (2010) [36]
MIROC-ESM	Tokyo, and National Institute for Environmental Studies (Japan)	128 × 65	Watanabe et al. (2011) [37]
MIROC-ESM-CHEM	Tokyo, and National Institute for Environmental Studies (Japan)	128 × 65	Watanabe et al. (2011) [38]
MPI-ESM-LR	Max Planck Institute for Meteorology, Germany	192 × 96	Block and Mauritsen. (2013) [39]
MRI-CGCM3	Meteorological Research Institute, Japan	320 × 160	Yukimoto et al. (2012) [40]
NorESM1-M	Norwegian Climate Centre, Norway	144 × 96	Bentsen et al. (2013) [41]

**Table 4:** The statistics of water balance and water suitability (1990~2013).

Sub-Region	W_mean_	RMSE_W_	EP_mean_	RMSE_EP_	U_mean_	RMSE_U_
Eastern	680.36 mm	70.10 mm	374.00 mm	71.11 mm	0.55	0.13
Western	603.65 mm	23.44 mm	374.26 mm	49.41 mm	0.62	0.10
Southern	713.35 mm	43.57 mm	420.88 mm	67.25 mm	0.59	0.15
Northern	722.82 mm	54.94 mm	361.41 mm	62.43 mm	0.50	0.13

Note: W_mean_ is the multi-year average water demand of apple plantations in the period of 1990–2013; RMSE_W_ is the root mean square error of the water demand series in the period of 1990–2013; EP_mean_ is the multi-year average effective precipitation in the period of 1990–2013; RMSE_EP_ is the root mean square error of the effective precipitation series in the period of 1990–2013; U_mean_ is the multi-year average water suitability in the period of 1990–2013; RMSE_U_ is the root mean square error of the water suitability series in the period of 1990–2013.

**Table 5:** The statistics of water balance and water suitability (2019~2050).

Sub-Region	W_mean_	RMSE_W_	EP_mean_	RMSE_EP_	U_mean_	RMSE_U_
Eastern	708.49 mm	20.78 mm	432.18 mm	36.70 mm	0.61	0.06
Western	704.52 mm	19.70 mm	408.61 mm	23.21 mm	0.58	0.04
Southern	698.74 mm	20.15 mm	440.21 mm	34.38 mm	0.63	0.06
Northern	734.42 mm	23.63 mm	418.62 mm	30.12 mm	0.57	0.05

Note: W_mean_ is multi-year average water demand of apple plantations in the period of 2019–2050; RMSE_W_ is the root mean square error of the water demand series in the period of 2019–2050; EP_mean_ is the multi–year average effective precipitation in the period of 2019–2050; RMSE_EP_ is the root mean square error of the effective precipitation series in the period of 2019–2050; U_mean_ is the multi–year average water suitability in the period of 2019–2050; RMSE_U_ is the root mean square error of the water suitability series in the period of 2019–2050.

**Table 6:** Contribution rate and sensitivity coefficient of four meteorological factors to $ET_{0}$.

	U_2_	R_n_	T_mean_	e_a_
Contribution rate	18%	36%	38%	8%
Sensitivity coefficient	0.27	0.46	0.34	−0.53
